# *MALAT1*/miR-15b-5p/*MAPK1* mediates endothelial progenitor cells autophagy and affects coronary atherosclerotic heart disease via mTOR signaling pathway

**DOI:** 10.18632/aging.101766

**Published:** 2019-02-21

**Authors:** Ying Zhu, Tianrui Yang, Jinlan Duan, Ninghui Mu, Tong Zhang

**Affiliations:** ^1^ Department of Geriatric & General Practice, The First People’s Hospital of Yunnan Province, The Affiliated Hospital of Kunming University of Science and Technology, Kunming 650032, Yunnan, China; ^*^ Equal contribution

**Keywords:** CAD, *MALAT1*, miR-15b-5p, *MAPK1*, mTOR signaling pathway

## Abstract

Objective: Present study focused on the influence of lncRNA *MALAT1* on coronary atherosclerotic heart disease (CAD) by regulating miR-15b-5p/*MAPK1* and mTOR signaling pathway.

Method: Differentially expressed genes and activated pathway were investigated through bioinformatics analysis. QRT-PCR was conducted to verify expression of *MALAT1*, miR-15b-5p and *MAPK1* in CAD blood samples and endothelial progenitor cells (EPCs). In addition, the interactions among *MALAT1*, miR-15b-5p and *MAPK1* were revealed by Luciferase reporter assay. Cell autophagy of EPCs was examined by Cyto-ID Autophagy Detection Kit and transmission electron microscope. MTT assay and flow cytometry were carried out to assess cell viability and apoptosis in different interference conditions. Western blot was performed to testify the expression of pERK1/2 (MAPK1), phosphorylated mTOR, ATG1 and LC3-II. Vascular cell adhesion molecule-1 (VCAM-1) and intercellular adhesion molecule-1 (ICAM-1) were detected by qRT-PCR. Finally, the effect of lncRNA *MALAT1* on cell autophagy and atherogenesis was tested *in vivo*.

Results: *MALAT1* was overexpressed in CAD blood samples and EPCs. Knockdown of *MALAT1* and *MAPK1* promoted cell viability, autophagy and further suppressed the development of CAD. Antago*MALAT1* protects mice against atherosclerosis.

Conclusion: LncRNA *MALAT1* inhibited EPCs autophagy and increased cell viability while repressed apoptosis of CAD via activating mTOR signaling pathway.

## INTRODUCTION

Coronary heart disease is primarily induced by atherosclerosis which is a systemic degenerative inflammatory vascular disease [1]. Coronary atherosclerotic heart disease (CAD) has a quite long history on its development, with a subclinical period. About half of all patients who die from coronary heart disease have no prior diagnosis or symptoms of cardiac disease [2]. During the several decades from 1900 to 1960, number of coronary heart disease deaths had a great increase, which obtained a close attention by many researchers. Some of them thought that the increase of incidence in coronary atherosclerosis is the key reason for the marked increase in deaths [3]. Autophagy is a lysosomal proteolytic mechanism designed to remove harmful proteins from cells and it is associated with maintaining a healthy state under stress [4]. Researches showed cell autophagy impairs atherosclerosis process while defective autophagy in cells enhances atherosclerosis [5, 6].

Human genome project illustrated that the majority of human genome could be transcribed to RNA positively, but there is only less than 2% of RNA which have ability to encode proteins [7]. Long non-coding RNA (lncRNA) is a form of non-coding RNAs, which have a length of more than 200 nucleotides [8]. Research showed that lncRNAs have an aberrant expression in some cancer tissues, and also they are usually associated with tumor suppressive or oncogenic processes [9]. As to the current research which shows that some lncRNAs are able to regulate the transcription of neighboring genes with an apparent cis-acting mechanism [10]. In recent years, lncRNA appears as an important regulatory factor in the pathogenesis of atherosclerosis [11, 12]. Emerging evidences suggested that lncRNAs are involved in the regulation of autophagy progression. The realization of most regulation depends on the effect of lncRNAs on key genes [13–15]. However, the effect of lncRNAs on atherosclerosis by regulating autophagy remains to be explored.

Metastasis-associated lung adenocarcinoma transcript 1 (*MALAT1*) is situated in chromosome 11 (11q13.1), with a length of exceeding 8000 nucleotides. And it has been well-studied in the field of biology, as one of lncRNAs [16, 17]. *MALAT1* has vital functions in nuclear speckles and regulation of genes expressions [18]. Furthermore, it has an underlying effect on the regulation of alternative splicing and cell cycle [19, 20]. Recent studies have identified that *MALAT1* was overexpressed and oncogenic in some tumors, including lung, colorectal, bladder and laryngeal cancers [21–23]. The role that *MALAT1* played in cardiovascular disease was also explored. Katharina *et al.* found that hypoxia decreased *MALAT1* in endothelial cells and inhibited endothelial cell proliferation [24]. *MALAT1* induces CD36 expression so that enhances lipid uptake in macrophages, accelerates cholesterol-filled foam cell accumulation in blood vessels. Subsequently, the apoptosis of foam cell promotes atherosclerosis process [25].

MicroRNAs (miRNAs) have a short length of 19–24 nucleotides, which could regulate genes expression post-transcriptionally. Commonly, they have two approaches to realize the function of preventing or changing production of the protein product, one is combination with complementary target sequences in mRNA, the other one is intervention with the translational machinery [26]. According to results of bioinformatics and cloning studies, researchers found that about 50 circulating miRNAs related to cardiovascular diseases [27]. Many researches have proved that miRNAs (miR-1, miR133a, miR-133b) play an important role in cardiac damage and myocardial infarction [26]. What’s more, some studies pay attention to the direction that circulating miRNAs have an effect of diagnostic and prognostic biomarkers [28]. Cenarro *et al.* revealed exposure to atherogenic lipoproteins modified the miRNA profile of coronary artery smooth muscle cells (CASMC) derived microvesicles including miR-15b-5p [29]. Besides, mitogen-activated protein kinase 1 (*MAPK1*), as one of mRNAs, is a candidate target gene for several miRNAs [30]. Researches shown that miR-197 led to silencing of the *MAPK1* gene by recognizing and then specifically binding to the predicted site of the *MAPK1* mRNA 3’-untranslated region [31]. Several researches showed *MAPK1* played an important role in atherosclerotic lesions or process [32–34].

The mammalian target of rapamycin (mTOR) is a serine/threonine kinase which belongs to the PI3K-associated kinase family. Besides, mTOR could gather into two large form of protein complexes, mTOR complex 1 and mTOR complex 2 [35]. In addition, the two protein complexes were regulated independently by its associated partners [36, 37]. Previous study showed that inhibition of the mTOR pathway decreased lipid accumulation, mTOR pathway stimulated autophagy in macrophages and prevented atherosclerotic plaque formation [38–40].

Based on the analysis above, we deduced that there was a potential connection between lncRNA, miRNA and mRNA, for example, lncRNA could directly target miRNA, and mRNA is a functional target of miRNA. Totally, in this study, we try to reveal the effect of lncRNA *MALAT1* on EPCs autophagy to affect CAD progress by the way of regulating miR-15b-5p and its target gene *MAPK1* and mTOR pathway.

## RESULTS

### *MALAT1* and *MAPK1* were up-regulated in CAD blood samples

**Figure 1:**
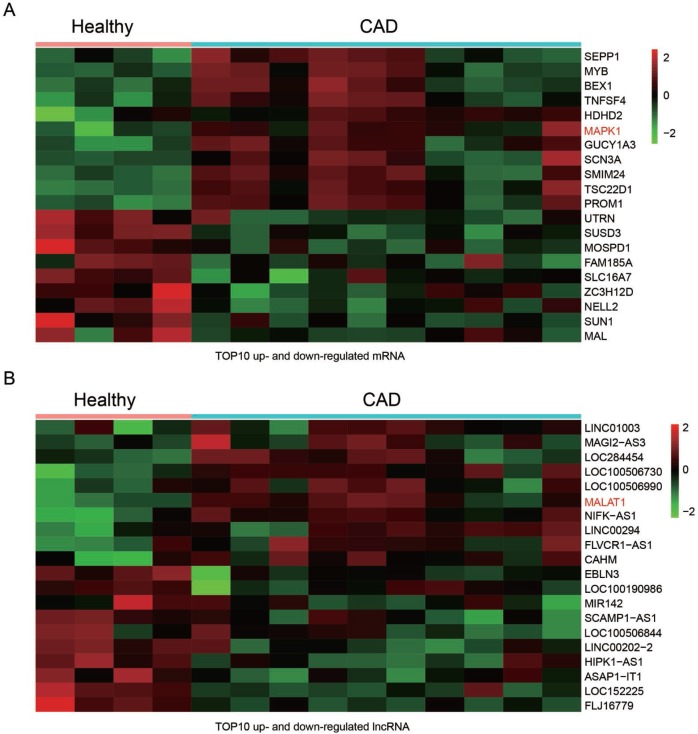
**Differentially expressed lncRNAs and mRNAs in CAD blood samples**. (**A**) Heat maps showed the 10 most up and down regulated mRNAs. MAPK1 was enhanced in CAD blood samples. (**B**) Heat maps showed the 10 most up and down regulated lncRNAs. LncRNA *MALAT1* was promoted in CAD blood samples.

The GSE18608 data was analyzed. There were 14 samples including 10 CAD blood samples (CAD group) and 4 healthy blood samples (Healthy group). The differentially expressed mRNAs and lncRNA were chosen under the screening norm of fold change (FC) >2 and *P* <0.05. Totally, 55 differentially expressed mRNAs and 108 differentially expressed lncRNAs were respectively identified. The top ten up and down-regulated mRNAs were selected to draw the cluster heat map (Figure 1A), including mRNA *MAPK1*. Similarly, the top ten differentially expressed lncRNAs were illustrated in heat map (Figure 1B). In addition, *MALAT1* was among the up-regulated lncRNAs.

### The GO pathways were detected by GSEA

**Figure 2:**
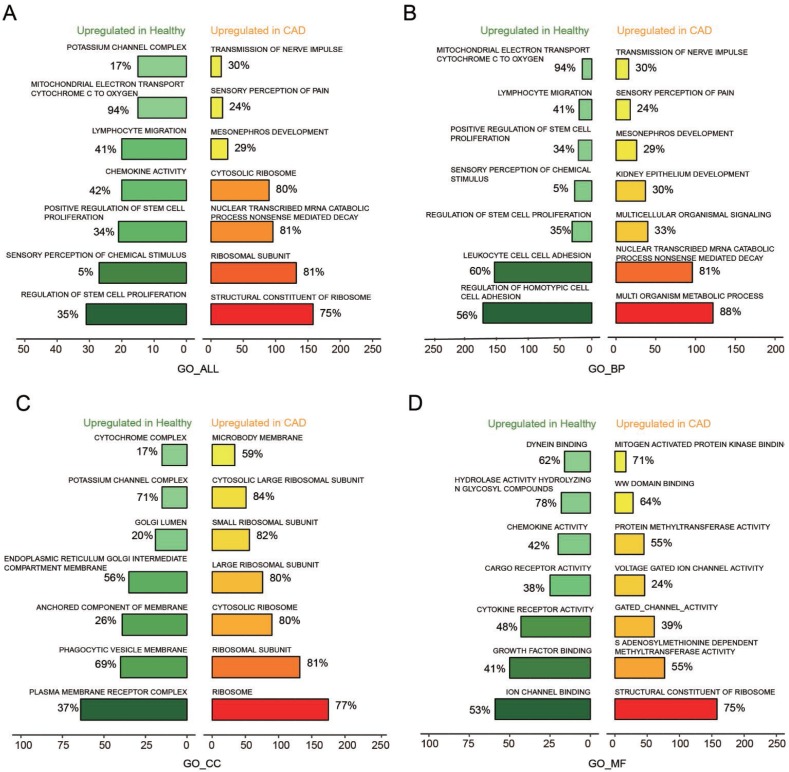
**Seven most significantly enriched pathways up-regulated in healthy and CAD blood samples were presented**. (**A**–**D**) Seven most distinctively activated GO_ALL, GO_BP, GO_CC, GO_MF pathways in healthy and CAD blood samples.

The key pathways that could affect CAD were testified through analysis of GO term enrichment, on the basis of chosen mRNAs that were differentially expressed. According to the enrichment result based on the GSEA reports, We illustrated top seven pathways after enrichment analysis with GO_Biological process (GO_BP), GO_Cellular Component (GO_CC), GO_Molecular Function (GO_MF) for further research on CAD (Figure 2A–2D). As to biological process, the up-regulated genes obtained a significant enrichment in transmission of nerve impulse, sensory perception of pain, mesonephros and kidney epithelium development, *etc* (Figure 2B). While for cellular component, the over-expressed genes enriched significantly in microbody membrane, large ribosomal subunit, ribosome, cytosolic ribosome,* etc* (Figure 2C). In regards to molecular function, the up-regulated genes obtained a significant enrichment in gated channel activity, structural constituent of ribosome, protein methyltransferase activity, *etc* (Figure 2D)*.*

### mTOR signaling pathway was activated in CAD

**Figure 3:**
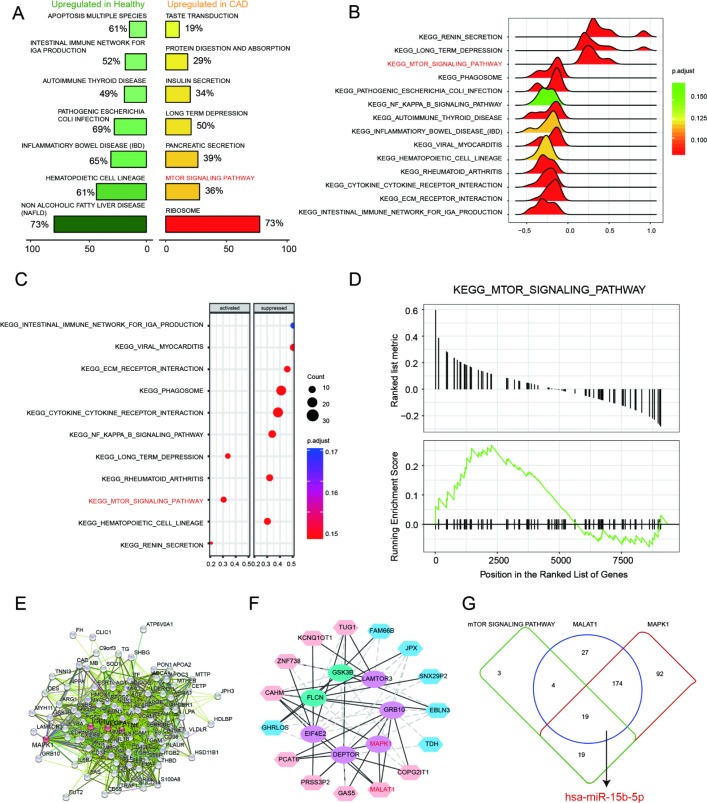
**mTOR signaling pathway and *MALAT1*/miR-15b-5p/*MAPK1* axis in CAD**. (**A**) Seven most distinctively activated KEGG pathways in healthy and CAD blood samples. (**B**–**C**) Dotplot and joyplot suggested the distributions of some KEGG pathways gene sets in all differential genes. (**D**) GSEA enrichment plot shows most related genes of mTOR signaling pathway are discovered in the region where genes are overexpressed in CAD. (**E**) Networks for CAD related mRNAs and the red ranked mRNAs are in KEGG_MTOR_Signaling_Pathway, including *MAPK1*. (**F**) The interacted networks for differentially expressed lncRNAs and mRNAs. The solid line indicates positive correlation between lncRNA *MALAT1* and *MAPK1*. (**G**) Venn diagram revealed that miR-15b-5p was one of the miRNAs not only targeted by lncRNA *MALAT1* and *MAPK1* but also involved in mTOR signaling pathway.

Based on the enrichment result from GSEA report, top 7 scored KEGG pathways in healthy or CAD blood samples were illustrated in Figure 3A. The over-expressed genes enriched in taste transduction, protein digestion and absorption, insulin secretion, long term depression, pancreatic secretion, mTOR signaling pathway and ribosome. We discovered that mTOR signaling pathway was activated in CAD blood samples. In addition, 14 KEGG pathways were observed in joy-plot, including 3 suppressed pathways and 11 activated pathways, and mTOR signaling pathway belonged to those activated pathways (Figure 3B). The dot-plot of KEGG pathway also illustrated that mTOR signaling pathway was one of the three pathways activated in CAD blood samples (Figure signaling pathway belonged to those activated pathways (Figure 3B). The dot-plot of KEGG pathway also illustrated that mTOR signaling pathway was one of the three pathways activated in CAD blood samples (Figure 3C). Further, GSEA enrichment result illustrated that most of genes, associated with mTOR signaling pathway, were revealed in the region where genes were enhanced in CAD (Figure 3D), which indicated that mTOR signaling pathway was activated in CAD.

### *MALAT1*/miR-15b-5p/*MAPK1* signal axis

On the basis of STRING database, interactions between CAD-related genes and the differentially expressed mRNAs in CAD were explored systematically. The consequence showed that the up-regulated mRNA *MAPK1* was not only relevant to CAD but also involved in mTOR signaling pathway (Figure 3E). Further, the interaction network between differentially expressed lncRNAs and mRNAs was drawn by Cytoscape and exhibited in Figure 3F. It revealed that lncRNA *MALAT1* was positively associated with *MAPK1*. There were 45 miRNAs contained in mTOR signaling pathway whereas 224 miRNAs and 304 miRNAs were respectively targeted by lncRNA *MALAT1* and *MAPK1*. Venn diagram was used to fine the intersection miRNAs and miR-15b-5p was one of them. Therefore, *MALAT1*/miR-15b-5p/*MAPK1* might be a signal axis and would be verified in the subsequent experiments (Figure 3G).

### LncRNA *MALAT1* inhibits cell autophagy and promotes CAD progression

**Figure 4:**
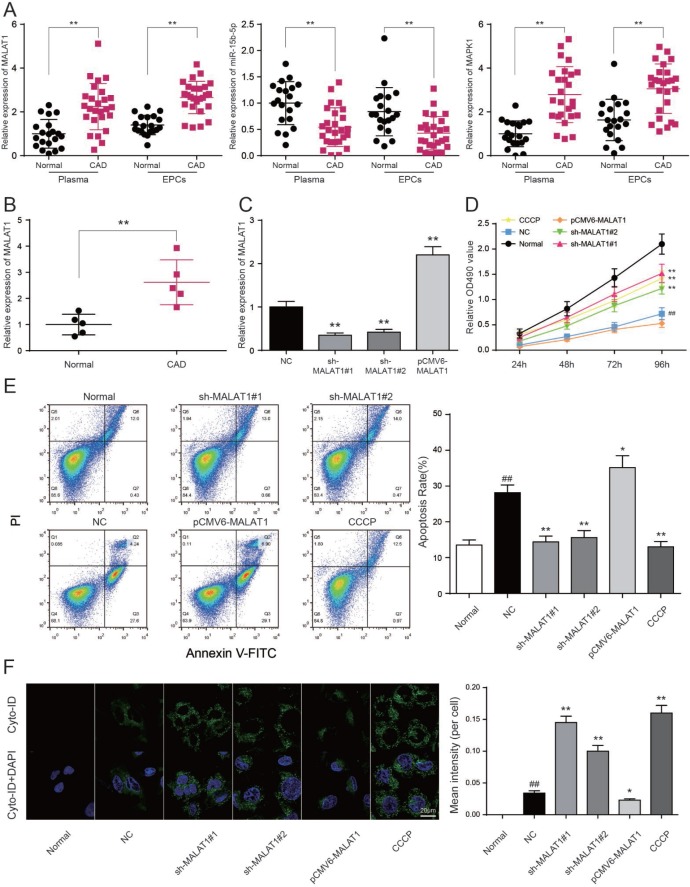
**LncRNA *MALAT1* inhibits cell autophagy and promotes CAD progression**. (**A**) *MALAT1* and *MAPK1* were overexpressed in CAD blood samples and EPCs while miR-15b-5p was down-regulated in CAD blood samples and EPCs. **P<0.01, compared with normal (healthy) group. (**B**) *MALAT1* expression in 5 CAD EPC samples was higher than that in 5 healthy EPC samples. **P<0.01, compared with normal group. (**C**) *MALAT1* was depressed in EPCs transfected with sh-*MALAT1*#1 or sh-*MALAT1*#2 detected by qRT-PCR. **P<0.01, compared with NC group. (**D**) MTT results showed that cell viability was promoted in EPCs transfected with sh-*MALAT1*#1 or sh-*MALAT1*#2. **P<0.01, compared with NC group; ##*P*<0.01, compared with normal group. (**E**) FCM results revealed that sh-*MALAT1*#1 or sh-*MALAT1*#2 restrained cell apoptosis rate of EPCs and there was significant difference between normal group and NC group. *P<0.05, **P<0.01, compared with NC group; ##*P*<0.01, compared with normal group. (**F**) Autophagy assay results revealed that sh-*MALAT1*#1, sh-*MALAT1*#2 and CCCP raised EPCs autophagy rate and there was conspicuous difference between normal group and NC group. *P<0.05, **P<0.01, compared with NC group; ##*P*<0.01, compared with normal group.

**Figure 5:**
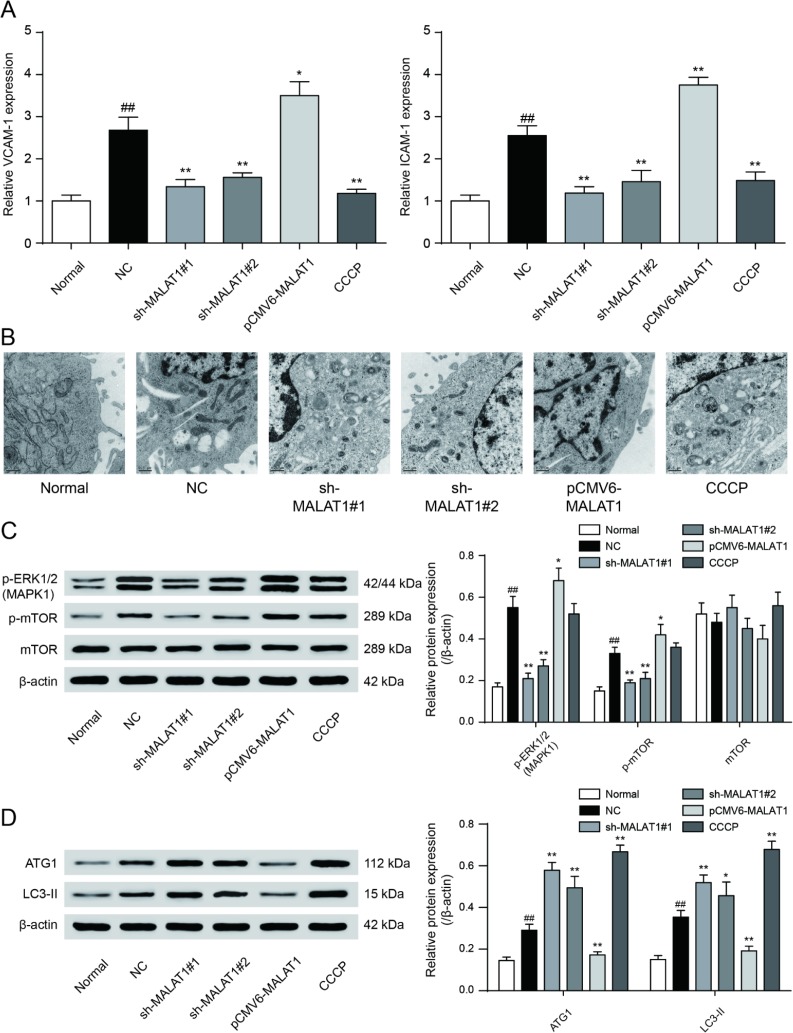
**VCAM-1/ICAM-1 expression and mTOR signaling pathway expression in cells**. (**A**) QRT-PCR results exhibited that sh-*MALAT1*#1, sh-*MALAT1*#2 and CCCP suppressed VCAM-1 and ICAM-1 expression and there was conspicuous difference between normal group and NC group, knockdown of *MALAT1* could inhibit CAD progress effectively. *P<0.05, **P<0.01, compared with NC group; ##*P*<0.01, compared with normal group. (**B**) Transmission electron microscope showed the autophagosome in sh-MALAT1#1 or, sh-MALAT1#2 and CCCP group was more than in NC group. (**C**) mTOR signaling pathway expression in different transfection groups. *P<0.05, **P<0.01, compared with NC group; ##*P*<0.01, compared with normal group. (**D**) ATG1 and LC3-II expression level in different transfection groups. *P*<0.05, **P<0.01, compared with NC group; ##*P*<0.01, compared with normal group.

To testify the expression of *MALAT1*, miR-15b-5p and *MAPK1*, qRT-PCR was performed and the results illustrated that *MALAT1* and *MAPK1* expression in CAD blood samples and EPCs were significantly up-regulated while miR-15b-5p was conspicuously down-regulated (*P*<0.01, Figure 4A). Then we selected 5 CAD samples and 5 healthy samples (EPCs) for further experiments. Similarly, *MALAT1* expression was significantly up-regulated in these 5 EPC samples from CAD patients than that in 5 EPC samples form healthy people (*P*<0.01, Figure 4B). EPCs transfected with either sh-*MALAT1*#1 or sh-*MALAT1*#2 exhibited lower level of expression compared to NC group while pCMV6-MALAT1 group had a higher expression level (*P*<0.01, Figure 4C). And expression level of sh-*MALAT1*#1 group was even lower than sh-*MALAT1*#2 group. MTT assay revealed that sh-*MALAT1*#1 promoted cell viability of CAD EPCs obviously whereas sh-*MALAT1*#2 and CCCP also facilitated cell viability (*P*<0.01, Figure 4D). Simultaneously, the results showed that there was conspicuous difference of cell viability between normal (healthy EPCs) and NC (CAD EPCs) groups (*P*<0.01, Figure 4D). Results of FCM assay verified that apoptosis rate in NC group was higher than that in normal group (*P*<0.05, Figure 5E), sh-*MALAT1*#1, sh-*MALAT1*#2 and CCCP had an effect of depression on cell apoptosis rate in EPCs (*P*<0.01, Figure 4E). And sh-*MALAT1*#1 appeared to be more effective compared with sh-*MALAT1*#2. The apoptosis rate in pCMV6-*MALAT1* group was higher than that in NC group (*P*<0.05, Figure 4E). Autophagy assay exhibited that there was almost no autophagy in normal group. Sh-*MALAT1*#1, sh-*MALAT1*#2 and CCCP had an effect of promoting on cell autophagy in EPCs comparing with NC groups and sh-*MALAT1*#1 had a better effect than sh-*MALAT1*#2 (*P*<0.05, *P*<0.01, Figure 4F). CAD markers VCAM-1 and ICAM-1 [41, 42] were detected by qRT-PCR and the results showed VCAM-1 and ICAM-1 expression in NC group were higher than that in normal group. Besides, sh-*MALAT1*#1, sh-*MALAT1*#2 and CCCP could significantly down-regulate VCAM-1/ICAM-1 expression (*P*<0.05, *P*<0.01, Figure 5A). Transmission electron microscope assay showed that autophagosome in sh-*MALAT1*#1 or, sh-*MALAT1*#2 and CCCP group was more than in NC group (Figure 5B). To determine whether *MALAT1* can activate mTOR signaling, we first detected the upstream modulators ERK1/2 under different conditions by western blot. Western blot results exhibited p-ERK1/2 (MAPK1), p-mTOR expressions were promoted by pCMV6-*MALAT1* while they were inhibited by sh-*MALAT1*#1 or sh-*MALAT1*#2. There was no significant change between CCCP group and NC group (*P*<0.05, *P*<0.01, Figure 5C). In addition, we detected ATG1 which was down-stream protein of mTOR and related to autophagy process. Furthermore, autophagy positive marker LC3-II was also detected. The result showed that ATG1 and LC3-II were highly expressed in sh-MALAT1#1, sh-MALAT1#2 and CCCP group while lowly expressed in pCMV6-*MALAT1* group (*P*<0.05, *P*<0.01, Figure 5D). Therefore, sh-*MALAT1*#1 was chosen to be used in the following experiments. Consequently, both sh-*MALAT1*#1 and sh-*MALAT1*#2 can facilitate cell viability and cell autophagy while restrain cell apoptosis. In other words, lncRNA *MALAT1* strengthens CAD progression.

### LncRNA *MALAT1* directly targets miR-15b-5p to promote CAD progression

**Figure 6:**
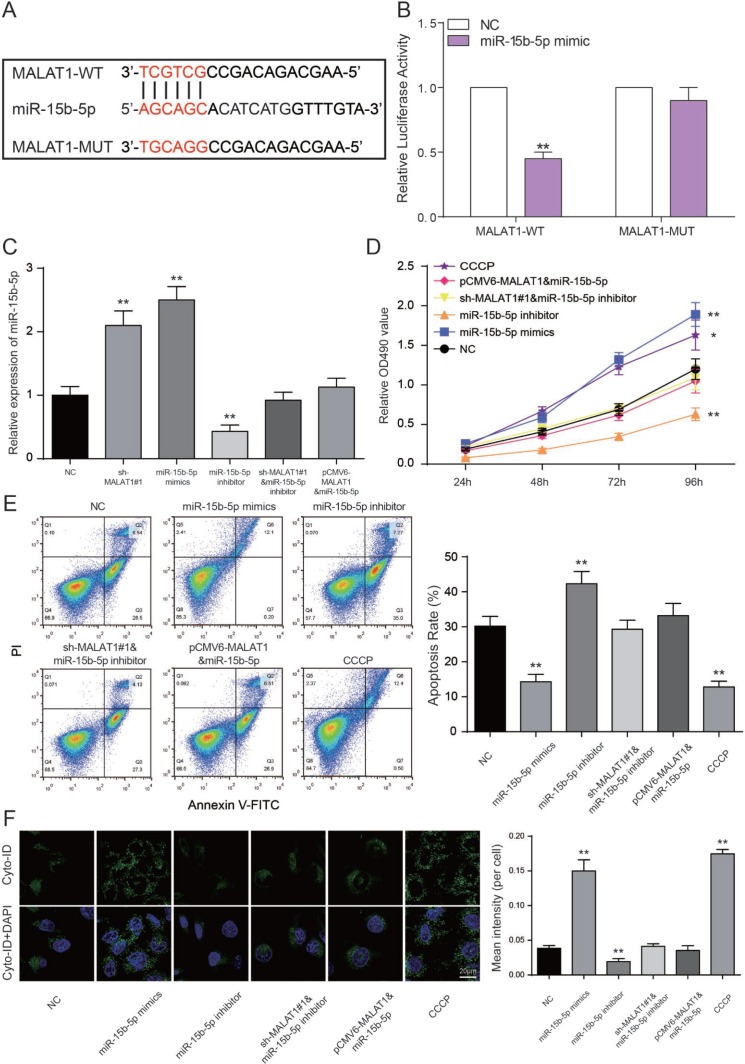
**LncRNA *MALAT1* directly targets miR-15b-5p to promote CAD progression**. (**A**) The predicted binding site. (**B**) Luciferase activity was declined notably when HEK 293T cells were co-transfected with *MALAT1* wild type and miR-15b-5p mimics. **P<0.01, compared with NC group. (**C**) Expression of miR-15b-5p was increased in both miR-15b-5p mimics and sh-*MALAT1*#1 groups whereas down-regulated in miR-15b-5p inhibitor group. MiR-15b-5p inhibitor + sh-*MALAT1*#1 group and miR-15b-5p mimics + pCMV6-*MALAT1* were consistent with NC group. **P<0.01, compared with NC group. (**D**) MTT results illustrated that miR-15b-5p mimics and CCCP strengthened cell viability of EPCs and miR-15b-5p inhibitor suppressed cell viability of EPCs. Co-transfection of miR-15b-5p inhibitor and sh-*MALAT1*#1 / miR-15b-5p mimics and pCMV6-*MALAT1* led to similar results of the NC group. *P<0.05, **P<0.01, compared with NC group. (**E**) FCM results revealed that both miR-15b-5p mimics and CCCP repressed cell apoptosis rate of EPCs while miR-15b-5p inhibitor promoted apoptosis rate. Co-transfection of miR-15b-5p inhibitor and sh-*MALAT1*#1 / miR-15b-5p mimics and pCMV6-*MALAT1* had little influence on cell apoptosis rate of EPCs. **P<0.01, compared with NC group. (**F**) Autophagy assay revealed that both miR-15b-5p mimics and CCCP promoted EPCs autophagy while miR-15b-5p inhibitor repressed cell autophagy. Co-transfection of miR-15b-5p inhibitor and sh-*MALAT1*#1 / miR-15b-5p mimics and pCMV6-*MALAT1* had little influence on cell autophagy. **P<0.01, compared with NC group.

The binding site between *MALAT1* and miR-15b-5p was shown in Figure 6A. We performed a luciferase reporter assay to validate the transcriptional regulation of miR-15b-5p on *MALAT1*. We found that co-transfection of *MALAT1* wild type and miR-15b-5p mimics decreased luciferase activity as compared with miR-NC group and *MALAT1* mutant type group (*P*<0.01, Figure 6B). The results indicated that miR-15b-5p served as a target of *MALAT1*. Furthermore, miR-15b-5p expressions of sh-*MALAT1*#1 group and miR-15b-5p mimics group were obviously raised while low expression was detected in the miR-15b-5p inhibitor group. MiR-15b-5p inhibitor + sh-*MALAT1*#1 group and miR-15b-5p + pCMV6-*MALAT1* group presented the same expression level as NC group (*P*<0.01, Figure 6C). After MTT assay, cell viabilities of CCCP group and miR-15b-5p mimics group were enhanced. In contrast, miR-15b-5p inhibitor group was suppressed. Meanwhile, cell viabilities of miR-15b-5p inhibitor + sh-*MALAT1*#1 group and miR-15b-5p + pCMV6-*MALAT1* group were practically equal to NC group (*P*<0.05, *P*<0.01, Figure 6D). The results of FCM assay proved that the rate of cell apoptosis in CCCP group and miR-15b-5p mimics group were remarkably lower than other groups. Apoptosis rate in miR-15b-5p inhibitor group was the highest and the one in miR-15b-5p inhibitor + sh-*MALAT1*#1 group and miR-15b-5p + pCMV6-*MALAT1* group was practically equivalent to NC group (*P*<0.01, Figure 6E). The results of autophagy assay proved that the cell autophagy in CCCP group and miR-15b-5p mimics group were remarkably higher than other groups. Autophagy in miR-15b-5p inhibitor group was the lowest and the one in miR-15b-5p inhibitor + sh-*MALAT1*#1 group and miR-15b-5p + pCMV6-*MALAT1* group was practically equivalent to NC group (*P*<0.01, Figure 6F). Based on the experiments mentioned above, lncRNA *MALAT1* can target miR-15b-5p to promote cell apoptosis and suppress the cell autophagy.

### *MAPK1* as a functional target of miR-15b-5p regulates CAD progression

**Figure 7:**
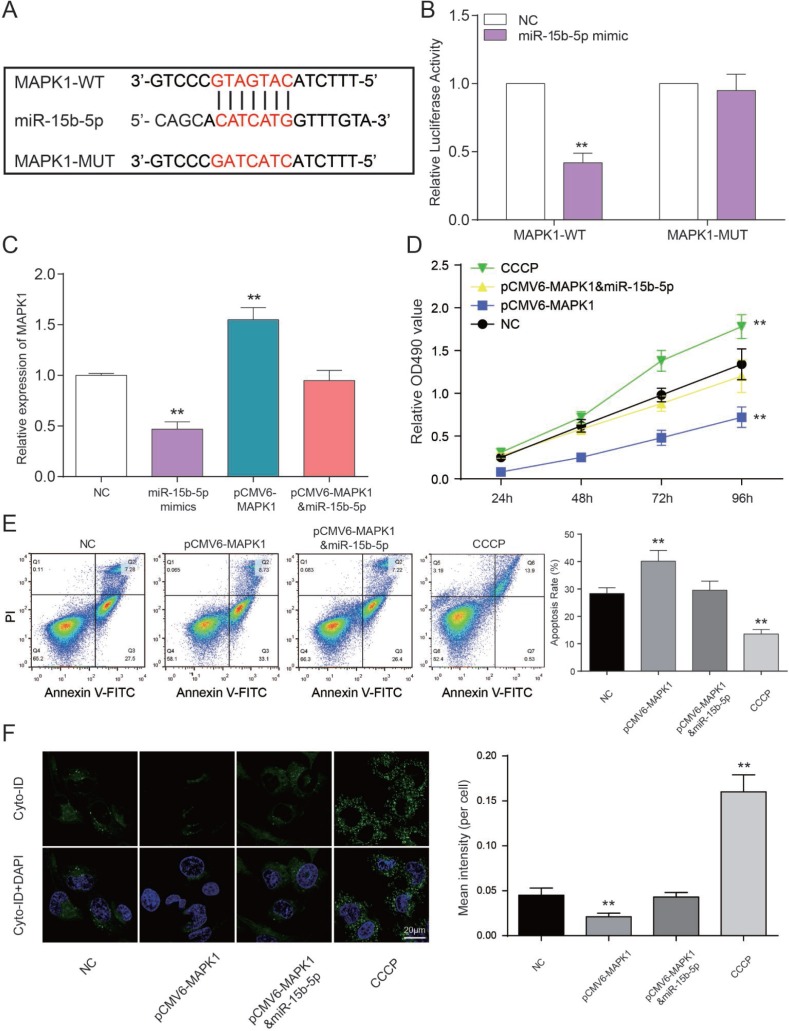
***MAPK1* as a functional target of miR-15b-5p regulates CAD progression**. (**A**) The predicted binding site. (**B**) Co-transfection of *MAPK1* wild type and miR-15b-5p mimics decreased luciferase activity. **P<0.01, compared with NC group. (**C**) *MAPK1* was down-regulated by CCCP while up-regulated by pCMV6-*MAPK1*. MiR-15b-5p mimics + pCMV6-*MAPK1* group was aligned with NC group. **P<0.01, compared with NC group. (**D**) MTT results demonstrated that cell viability of EPCs were promoted by CCCP and inhibited by pCMV6-*MAPK1*. MiR-15b-5p mimics + pCMV6-*MAPK1* group had almost no influence on cell viability of EPCs. **P<0.01, compared with NC group. (**E**) FCM results illustrated that CCCP group presented declined apoptosis rate of EPCs while pCMV6-*MAPK1* group presented increased apoptosis rate. MiR-15b-5p mimics + pCMV6-*MAPK1* group presented the same apoptosis rate as NC group. **P<0.01, compared with NC group. (**F**) Autophagy results illustrated that CCCP group accelerated EPCs autophagy while pCMV6-*MAPK1* group presented declined autophagy. MiR-15b-5p mimics + pCMV6-*MAPK1* group presented the same autophagy as NC group. **P<0.01, compared with NC group.

The binding site between *MAPK1* and miR-15b-5p was shown in Figure 7A. Luciferase reporter assay was performed to explore the interaction between miR-15b-5p and *MAPK1*. The intensity of luciferase in HEK 293T cells co-transfected with* MAPK1* wild type and miR-15b-5p mimics was remarkably reduced, while the type that are *MAPK1* mutant had no obvious fluctuation (*P*<0.01, Figure 7B). Therefore, miR-15b-5p can directly target to *MAPK1*. In addition, expression level of *MAPK1* in miR-15b-5p mimics group was notably declined while pCMV6-*MAPK1* group was promoted according to the results of qRT-PCR. And *MAPK1* expression level of miR-15b-5p mimics + pCMV6-*MAPK1* group and NC group were approximately equal (*P*<0.01, Figure 7C). MTT assay illustrated that CCCP group emerged the highest cell viability. Nevertheless, pCMV6-*MAPK1* group was significantly declined. And there’s nearly no difference between miR-15b-5p mimics + pCMV6-*MAPK1* group and NC group (*P*<0.01, Figure 7D). On the basis of FCM assay, rate of cell apoptosis in CCCP group was suppressed and it was enhanced in pCMV6-*MAPK1* group. Simultaneously, rate of cell apoptosis in miR-15b-5p mimics + pCMV6-*MAPK1* group was still the same as NC group (*P*<0.01, Figure 7E). The results of autophagy assay proved that the cell autophagy in CCCP group were remarkably higher than other three groups. Autophagy in pCMV6-*MAPK1* group was lower than others and the one in miR-15b-5p mimics + pCMV6-*MAPK1* group was practically equivalent to NC group (*P*<0.01, Figure 7F). Taken together, miR-15b-5p represses *MAPK1* to regulate cell viability and apoptosis rate and autophagy to affect CAD progress.

### LncRNA *MALAT1* enhanced CAD progression via activating mTOR signaling pathway

**Figure 8:**
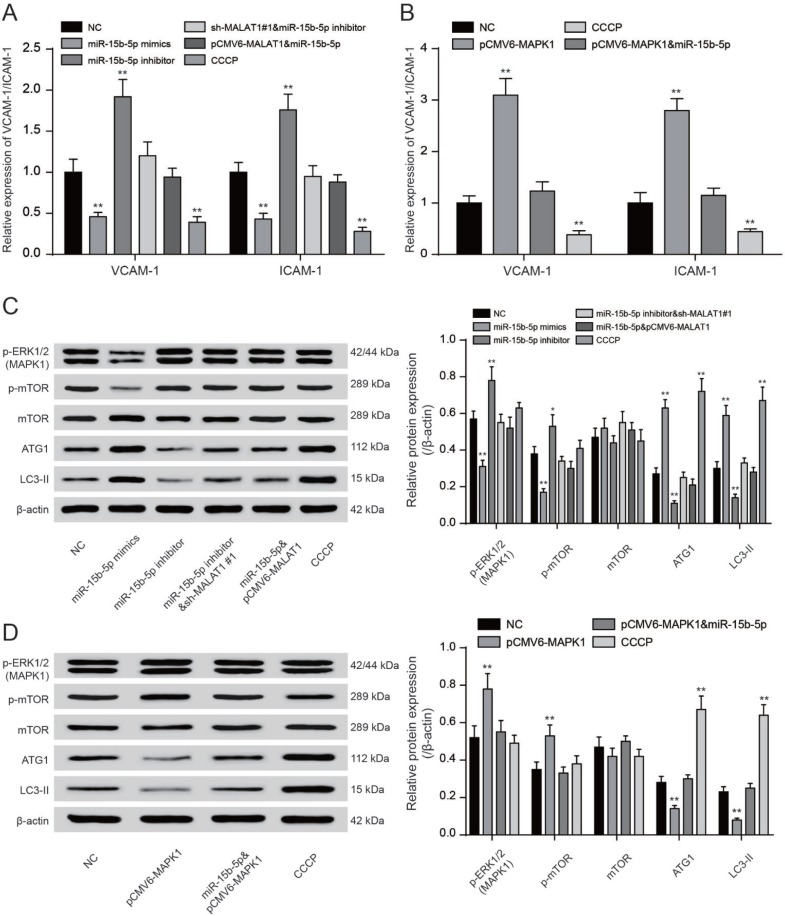
**LncRNA *MALAT1* enhanced CAD progression via activating mTOR signaling pathway**. (**A**,** B**) QRT-PCR results of VCAM-1/ICAM-1 expression in different transcription groups. **P<0.01, compared with NC group. (**C, D**) Western blot results showed that both sh-*MALAT1*#1 and miR-15b-5p mimics depressed expression of ERK1/2, phosphorylated mTOR. Both CCCP and miR-15b-5p mimics promoted expression of ATG1 and LC3-II. Nevertheless, both miR-15b-5p inhibitor and pCMV6-*MAPK1* suppressed expression of ATG1 and LC3-II. Nevertheless, both miR-15b-5p inhibitor and pCMV6-*MAPK1* enhanced expression of *MPKA1* and phosphorylated mTOR. MiR-15b-5p inhibitor + sh-*MALAT1*#1 group, miR-15b-5p mimics + pCMV6-*MALAT1* as well as miR-15b-5p mimics + pCMV6-*MAPK1* group exhibited similar results of NC group. *P<0.05, **P<0.01, compared with NC group.

QRT-PCR showed that CCCP and miR-15b-5p mimics could significantly reduce VCAM-1/ICAM-1 expression while miR-15b-5p inhibitor and pCMV6-*MAPK1* could promote VCAM-1/ICAM-1 expression (*P*<0.01, Figure 8A, 8B). MiR-15b-5p mimics could significantly reduce VCAM-1/ICAM-1 expression while the inhibiting effect could be offset by pCMV6-*MALAT1* or pCMV6-MAPK1. MiR-15b-5p inhibitor could conspicuously increase VCAM-1/ICAM-1 expression while the promoting effect could be counteracted by sh-*MALAT1*#1 (*P*<0.01, Figure 8A, 8B). The levels of phosphorylated ERK1/2 were added in EPCs transfected with miR-15b-5p inhibitor or pCMV6-*MAPK1* while reduced in EPCs transfected with sh-*MALAT1*#1 or miR-15b-5p mimics. Meanwhile, co-transfection of miR-15b-5p inhibitor and sh-*MALAT1*#1 or co-transfection of miR-15b-5p mimics and pCMV6-MAPK1 were consistent with NC group (*P*<0.01, Figure 8C). We next investigated the phosphorylation of mTOR at Ser2448 and the total mTOR. The phosphorylation of mTOR was strongly enhanced in both miR-15b-5p inhibitor and pCMV6-*MAPK1* groups while CCCP and miR-15b-5p mimics groups obtained opposite results according to results of western blot. Meanwhile, we observed that the miR-15b-5p inhibitor + sh-*MALAT1*#1 group, miR-15b-5p mimics + pCMV6-*MALAT1* as well as miR-15b-5p mimics + pCMV6-*MAPK1* group were practically the same as the NC group (*P*<0.05, *P*<0.01, Figure 8C–8D). The western blot results showed that ATG1/LC3-II were strongly weaken in both miR-15b-5p inhibitor and pCMV6-MAPK1 groups while sh-*MALAT1*#1, CCCP and miR-15b-5p mimics groups obtained opposite results. Meanwhile, we observed that the miR-15b-5p inhibitor + sh-*MALAT1*#1 group, miR-15b-5p mimics + pCMV6-*MALAT1* as well as miR-15b-5p mimics + pCMV6-MAPK1 group were practically the same as the NC group (*P*<0.05, *P*<0.01, Figure 8C–8D). Overall, lncRNA *MALAT1* triggered mTOR signaling pathway via regulating miR-15b-5p and *MAPK1*.

### Antgo*MALAT1* protects mice against atherosclerosis

**Figure 9:**
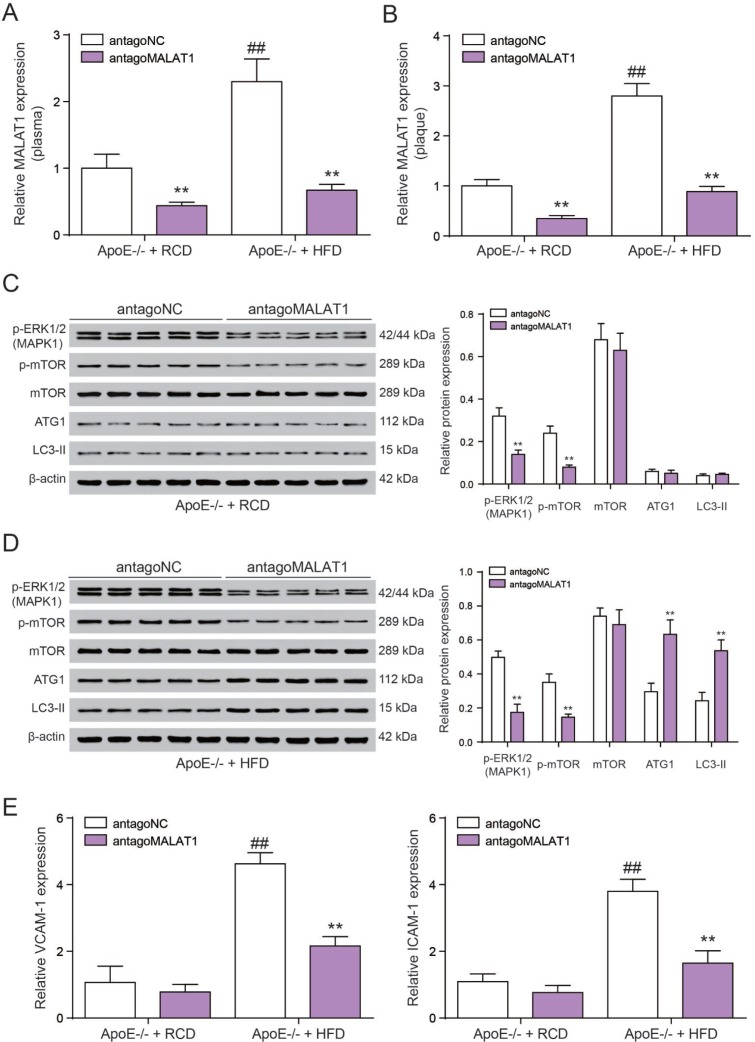
**Antgo*MALAT1* protects mice against atherosclerosis**. (**A**,** B**) The expression of *MALAT1* was determined by qRT-PCR in plasma and aortic tissue from ApoE−/− +RCD and ApoE−/− +HFD mice which were injected with antago*MALAT1* or antagoNC, n=5. **P<0.01, compared with antagoNC group; ##*P*<0.01, compared with ApoE−/− +RCD group. (**C**) Western blot result of MAPK1, phosphorylated mTOR, mTOR and autophagy related protein ATG1 and LC3-II in ApoE−/− +RCD group. **P<0.01, compared with antagoNC group. (**D**) Western blot result of MAPK1, phosphorylated mTOR, mTOR and autophagy related protein ATG1 and LC3-II in ApoE−/− +HFD group. **P<0.01, compared with antagoNC group. (**E**) QRT-PCR results of VCAM-1/ICAM-1 expression with the different treatment in ApoE−/− +RCD and ApoE−/− +HFD groups. **P<0.01, compared with antagoNC group; ##*P*<0.01, compared with ApoE−/− +RCD group.

**Figure 10:**
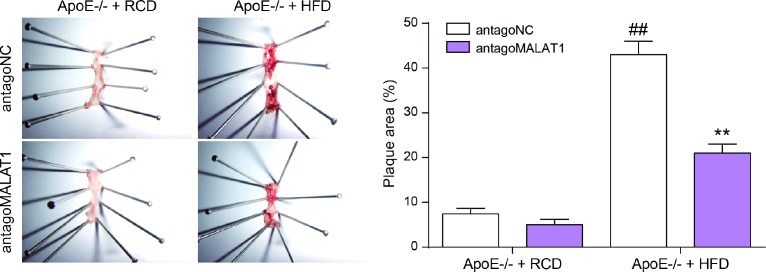
**En face analysis of thoracoabdominal aorta and cross-sections of the root of aorta**. The atherosclerotic lesions throughout the aorta in ApoE−/− mice were lessened in the antagoMALAT1 group and the plaque area in ApoE−/− +HFD group was larger than that in ApoE−/− +RCD group. **P<0.01, compared with antagoNC group. ##*P*<0.01, compared with ApoE−/− + RCD group.

Finally, we tested the role of lncRNA *MALAT1* on atherosclerosis *in vivo*. Six-week-old male ApoE^−/−^ mice were fed with HFD21 and RCD, and then injected with antago*MALAT1* or control through tail vein. In different feeding groups, the expression level of *MALAT1* in plasma and aortic tissue from antago*MALAT1*-injected ApoE^−/−^mice was descended sharply compared to NC group (*P*<0.01, Figure 9A, 9B). Western blot assay showed that MAPK1 and p-mOTR protein expression was significantly reduced by antago*MALAT1* in ApoE−/− + RCD group and in ApoE−/− + HFD group. Low expression of ATG1 and LC3-II reflected there was almost no autophagy in ApoE−/− + RCD group. Besides, ATG1 and LC3-II were conspicuously up-regulated when the *MALAT1* was knocked down in ApoE−/− + HFD group. Meanwhile, there was no significant change in mTOR expression (*P*<0.01, Figure 9C, 9D). Vascular cell adhesion molecule-1 (VCAM-1) and intercellular adhesion molecule-1 (ICAM-1) were highly expressed in ApoE−/− + HFD group. With the *MALAT1* knocking down, their expression was dramatically down-regulated (*P*<0.01, Figrure 9E). Based on an *en face* analysis of thoracoabdominal aorta and cross-sections of the root of aorta, the atherosclerotic lesions throughout the aorta in ApoE−/− mice were lessened in the antagoMALAT1 group and the plaque area in ApoE−/− +HFD group was larger than that in ApoE−/− +RCD group (*P*<0.01, Figure 10). As a result, lncRNA *MALAT1* inhibition attenuates atherosclerosis in mice.

## DISCUSSION

In this study, the GSE18608 data analysis illustrated that *MALAT1* was up-regulated, which was one of the differentially expressed lncRNAs in CAD blood samples and EPCs. In addition, *MALAT1*/miR-15b-5p/*MAPK1* signal axis was revealed by luciferase reporter. Results showed that miR-15b-5p serves as a target of *MALAT1* and that miR-15b-5p can directly target to *MAPK1*. Furthermore, lncRNA *MALAT1* could activate mTOR signaling pathway and affect cell proliferation, apoptosis and autophagy to mediate CAD progress according to the results. Also, researchers found that antgo*MALAT1* can protect mice against atherosclerosis *in vivo*.

Microarrays analysis can assist us to obtain the potential differentially expressed genes and relevant pathways, helping us to explore the underlying molecular mechanisms [43, 44]. In this study, GSE18608 data was analyzed. The differentially expressed mRNAs and lncRNA were selected, and there were 55 differentially expressed mRNAs and 108 differentially expressed lncRNAs. Furthermore, *MAPK1* and *MALAT1* were both up-regulated in CAD. Among the differentially expressed genes and lncRNAs in CAD tissues which from microarray analysis, *MAPK1* and *MALAT1* were chosen for our research because they were found to be related to CAD in previous studies [45–47]. Furthermore, *MALAT1* may serve as potential biomarkers of atherosclerosis [48, 49]. MAPK pathways were found playing roles in stroke progression [50, 51]. MiR-15b-5p was the co-target of *MAPK1* and *MALAT1*, it is worthy to explore *MALAT1*/miR-15b-5p/*MAPK1* signal axis acting in CAD progression. Pathway analysis is an impactful ideal, which can find the interesting related genes and pathways from different databases [52, 53]. In this study, the GO annotation analysis was detected by GSEA. According to the result, we can find that mTOR signaling pathway was activated in CAD. STRING tool is a pre-computed global resource which could do a favor of investigating the connections between genes associated with disease and the target proteins [54, 55]. In this study, STRING database was performed to confirm the connections between CAD associated genes and the differentially expressed mRNAs, and find the key genes which were also related to mTOR signaling pathway. Results showed that *MALAT1*/miR-15b-5p/*MAPK1* might be a signal axis and would be verified in the subsequent experiments.

*MALAT1* has vital functions in nuclear speckles and regulation of genes expressions [16, 18]. Recent researches have proved that *MALAT1* was overexpressed and oncogenic in some tumors, including lung, colorectal, bladder and laryngeal cancers [21–23]. In this study, expression of *MALAT1* in CAD blood samples was significantly up-regulated according to the results of qRT-PCR. In other words, lncRNA *MALAT1* strengthens CAD progression. This result was consisted with some previous researches [46, 56, 57]. We performed a luciferase reporter assay to validate the transcriptional regulation of miR-15b-5p on *MALAT1*, and found that lncRNA *MALAT1* directly targets miR-15b-5p to promote CAD progression. Next, the interaction between miR-15b-5p and *MAPK1* was also detected, and results showed that *MAPK1* as a functional target of miR-15b-5p regulates CAD progression.

mTOR pathway regulates the cell growth which is associated with energy, nutrients, growth factors and other environmental conditions, and it plays a prominent role in cancer [58]. Recent studies illustrated that mTOR protein kinase is a kind of critical growth-control node, as a receiver of stimulatory signals from Ras and PI3K downstream [59]. In this study, to determine whether *MALAT1* can activate mTOR signaling, we first detected the upstream modulators ERK1/2 and tuberous sclerosis 2 (TSC-2) under different conditions by western blot. We discovered that *MALAT1*/miR-15b-5p/*MAPK1* affect mTOR signaling to mediate cell autophagy and further affect CAD progress. Results showed that lncRNA *MALAT1* triggered mTOR signaling pathway via regulating miR-15b-5p and* MAPK1*.

Animal experiments have significant implications on the field of biomedicine [60, 61]. In this study, we tested the influence of lncRNA *MALAT1* on atherosclerosis *in vivo*. Results illustrated that *MALAT1* expression in plasma and aortic tissue from antago*MALAT1*-injected ApoE^−/−^ mice was descended sharply compared with the NC group. Besides, western blot and qRT-PCR results showed that autophagy protein ATG1 and LC3-II was significantly up-regulated while CAD marker protein VCAM-1/ICAM-1 were conspicuously down-regulated with the *MALAT1* knocking down. As a result, lncRNA *MALAT1* inhibition attenuates atherosclerosis in mice.

However, some limitations should attract more attentions. For example, mTOR signaling pathway was not the only activated pathways in CAD blood samples according to the result of dot-plot of KEGG pathway. Although most genes of mTOR signaling pathway were discover in CAD, the other two pathways still need to be further researched later. Further *in vivo* experiments were needed.

As a conclusion, lncRNA *MALAT1* repressed cell viability and autophagy while increased apoptosis of CAD via activating mTOR signaling pathway.

## MATERIALS AND METHODS

### Microarray analysis

The microarray data GSE18608 were analyzed, which was from Gene expression omnibus (GEO, https://www.ncbi.nlm.nih.gov/ds/). There were 14 samples including 4 healthy blood samples and 10 CAD blood samples. Differentially expressed genes (DEGs) between CAD samples and healthy samples were dealt with R software. The differentially expressed mRNAs and lncRNAs were screened out under conditions of fold change (FC)>2 and *P*<0.05. And then, the expression data of total normalized mRNAs were uploaded to GSEA v3.0 software to conduct KEGG pathway and GO term enrichment analysis. Default weighted enrichment statistic was adapted to process data for 1000 times under the condition of *P* < 0.05 which was considered to be significantly enriched. Differentially regulated results of GSEA reports were screened out to undergo graphics processing with “ggplot2” package in R language. The genes associated with CAD were acquired from DigSee (Disease Gene Search Engine with Evidence Sentences, http://gcancer.org/digsee). Using STRING database (https://string-db.org), interactions between CAD associated genes and the differentially expressed mRNAs were investigated comprehensively. The key genes which were also involved in mTOR signaling pathway were also identified. R software was used to find out the connections between these differentially expressed lncRNAs and mRNAs. Then, we graphed the networks using Cytoscape software. Among these networks, nodes represented DEGs, and the edges stood for existence of co-expression. MiRcode (http://www.mircode.org/) was used to predict miRNAs targeted with lncRNAs and mRNAs. Intersection miRNA finding was performed on Venn 2.1 (http://bioinfogp.cnb.csic.es/tools/venny/).

### Blood sample collection

Samples of atherosclerotic peripheral blood were collected from 26 CAD patients while the healthy blood samples were obtained from 20 volunteers in The First People’s Hospital of Yunnan Province from October 2017 to December 2017, the healthy blood samples were sampled for study to serve as controls. The basic clinical characteristics of patients were quantified by testing the levels of total cholesterol, low-density lipoprotein (LDL) cholesterol, triglycerides. Specific patient characteristics were showed in Table 1. Written consents were authorized from all patients, and the study protocol obtained a permission from the Ethics Committee of The First People’s Hospital of Yunnan Province.

**Table 1:** Patient characteristics

Characteristics total cases		Non-CAD	CAD	*P* value
		N=20	N=26	
Age (years)				0.0231*
	≤60	16	11	
	>60	4	15	
Gender				0.1852
	Male	10	18	
	Female	10	8	
Smoke				0.4466
	Yes	4	9	
	No	16	17	
Obesity				0.0593
	Yes	0	5	
	No	20	21	
Hypertension				0.0026**
	Yes	2	15	
	No	18	11	
Dyslipidemia				0.0061**
	Yes	1	12	
	No	19	14	
Lipid profile				
	T-CHOL(mg/dL)	168.5±32.5	215.2±43.8	<0.001**
	TG (mg/dL)	62.9±44.2	123.0±68.5	0.0014**
	LDL-C(mg/dL)	117.5±47.2	136.3±12.9	0.0033**

CAD, coronary atherosclerotic heart disease; T-CHOL, total cholesterol; TG, triglyceride; LDL-C, low-density lipoprotein cholesterol;*P<0.05, **P<0.01; Chi-square test and unpaired t-test were used.

### Plasma collection and storage

Peripheral blood from patients and control group were collected in EDTA tubes and processed within 2 hours by centrifuging at 1,000 g at 4 °C for 10 minutes. Plasma was transferred to a fresh RNase/DNase-free 1.5 ml EP tube (Axygen, Tewksbury, MA, USA) and centrifuged at 16,000g at 4 °C for 10 minutes. The supernatant was transferred to another fresh RNase/DNase-free tubes and stored at -80 °C.

### Isolation and cultivation of (endothelial progenitor cells) EPCs

Peripheral blood mononuclear cells were isolated from the peripheral blood of patients with CAD and healthy donors using ficoll density gradient centrifugation. The cells were then cultured on fibronectin-coated six-well plates in endothelial basal medium (Cambrex, Walkersville, MD, USA) supplemented with vascular epidermal growth factor (Preprotech, Rocky Hill, NJ, USA), human recombinant long insulin-like growth factor-1, ascorbic acid, cortisol and 20% FBS (Hyclone, South Logan, UT, USA) at 37 °C in a 5% CO_2_ incubator. After 4 days, non-adherent cells were removed by washing with PBS. Adherent cells (attached early EPCs) were incubated in fresh medium every 3 days and were used for the subsequent experiments. These cells had elongated spindle-shape morphology and their phenotype was confirmed by assessing the surface markers with flow cytometry analysis (Becton Dickinson, Franklin Lakes, NJ, USA). FITC-conjugated antibodies of CD31, CD34 and CD45 (Abcam, Cambridge, MA, USA) were used [62].

### Cell transfection

The transfection experiment was performed at Day 5 after initial plating. The sequences were desired by Sangon Biotech (Shanghai, China). Negative control (NC), miR-15b-5p mimics, miR-15b-5p inhibitor, sh-*MALAT1*#1 (5’-GCAGCCCGAGACTTCTGTAAA-3’), sh-*MALAT1*#2 (5’-GCCCGAGACTTCTGTAAAGGA-3’), pCMV6-*MALAT1* or pCMV6-*MAPK1* were transfected into mixed cortical cultures using Lipofectamine^TM^ 3000 according to the manufacturer’s instructions. The transfected cells were collected after 48 h for further experiments. EPCs were generally assigned to different groups as follows: normal group (healthy EPCs), NC group (cells transfected with no sense oligonucleotide sequence), sh-*MALAT1*#1 group (cells transfected with sh-*MALAT1*#1), sh-*MALAT1*#2 group (cells transfected with sh-*MALAT1*#2), pCMV6-*MALAT1* group (cells transfected with pCMV6-*MALAT1*), CCCP group (Carbonyl cyanide 3-chlorophenylhydrazone, Sigma, Buenos Aires, Argentina), miR-15b-5p mimics group (cells transfected with miR-15b-5p mimics), miR-15b-5p inhibitor group (cells transfected with miR-15b-5p inhibitor), miR-15b-5p inhibitor + sh-*MALAT1*#1 group (cells co-transfected with miR-15b-5p inhibitor and sh-*MALAT1*#1), miR-15b-5p + pCMV6-*MALAT1* group (cells co-transfected with miR-15b-5p mimics and pCMV6-*MALAT1*), pCMV6-*MAPK1* group (cells transfected with pCMV6-*MAPK1*), miR-15b-5p mimics + pCMV6-MAPK1 group (cells transfected with miR-15b-5p mimics and pCMV6-*MAPK1*).

### QRT-PCR

Total RNA was extracted from EPCs using TRIzol (Invitrogen, Carlsbad, CA, USA) according to the manufacturer instructions, and then cDNA was generated from RNA using SuperScript III (Invitrogen). *MALAT1*, MiR-15b-5p, *MAPK1*, VCAM-1 and ICAM-1 expressions were evaluated using the SYBR green quantitative PCR kit (Takara, Tokyo, Japan) based on the manufacturer description. β-actin and RNU6B were performed as a control internally for mRNAs and miRNAs, respectively. QRT-PCR was performed on the ABI Prism 7500 Fast Sequence Detection System (Applied Biosystems). Levels of relative expression were calculated and quantified with the 2^-ΔΔCt^ method. Primers were exhibited in Table 2.

**Table 2:** Primer sequences for RT-PCR

Primer	Sequences
MALAT1 forward	5’-GCCTGGAAGCTGAAAAACGG-3’
MALAT1 reverse	5’-TGGAAAACGCCTCAATCCCA-3’
miR-15b-5p forward	5’-TAGCAGCACATCATGGTTTACA-3’
miR-15b-5p reverse	5’-TGCGTGTCGTGGAGTC-3’
MAPK1 forward	5’-CAGTTCTTGACCCCTGGTCC-3’
MAPK1 reverse	5’-TACATACTGCCGCAGGTCAC-3’
ICAM1 forward	5’-CAGTGACCATCTACAGCTTTCCGG-3’
ICAM1 reverse	5’-GCTGCTACCACAGTGATGATGACAA-3’
VCAM1 forward	5’-GATACAACCGTCTTGGTCAGCCC-3’
VCAM1 reverse	5’-CGCATCCTTCAACTGGGCCTT-3’
β-actin forward	5’-GATCATTGCTCCTCCTGAGC-3’
β-actin reverse	5’-ACTCCTGCTTGCTGATCCAC-3
U6 forward	5’-GCUUCGGCAGCACAUAUACUAAAAU-3’
U6 reverse	5’-CGCUUCACGAAUUUGCGUGUCAU-3’

### Western blot

Cellular extracts were lysed using the lysis buffer RIPA, which was purchased from KeyGen Biotech Co. Ltd (Nanjing, China), and supernatant was collected after centrifugation. Proteins were separated using sodium dodecyl sulfate-polyacrylamide gel electrophoresis (SDS-PAGE) and were blotted onto polyvinylidene difluoride membranes (Bio-Rad, USA). Then, membranes with isolated proteins were blocked for 1 h and detected using primary antibodies including anti-phosphor-ERK1/2 (MAPK1) (ab50011, pT185/pY187, 1:2000), anti-mTOR (ab2732, 1:2000), anti-phospho-mTOR (mTORC1) (ab137133, S2448, 1:1000), anti-ATG1 (ULK1) (ab167139, 1µg/ml), anti-LC3-II (LC3B) (ab48394, 1 µg/ml) and anti-β-actin (ab8227, 1:100) (Santa Cruz, USA) antibody at 4 °C overnight. After that, membranes were washed thrice by Tris-buffered saline with Tween 20 (TBST), and Goat Anti-Rabbit IgG H&L (HRP, 1:2000) were injected into the membranes, which were incubated for another 1 h. Finally, membranes were washed thrice again using TBST. Immunobinding signals were tested by the chemiluminescence reagent, which was purchased from KeyGen Biotech Co. Ltd. Relative protein expression was identified through densitometry analysis using the Image-Pro Plus Version 6.0 software and calculated based on the β-actin loading control.

### MTT assay

In brief, we cultured those cells in 96-well plates (10,000/well) for 3 days prior to the addition of MTT. These plates were further incubated for 4 h; subsequently, dimethyl sulfoxide (DMSO; 160 μL) was put into each of those wells and the plates were vortexed for 8 min. The optical density (OD) at 490 nm was measured using a micro-plate reader. The growth chart was mapped with OD on the Y axis and time interval as the X axis. The cell viability was subsequently detected using the MTT method.

### Flow cytometry analysis (FCM)

Cell apoptosis pattern was evaluated by the Annexin V-FITC Apoptosis Detection Kit. EPCs were seeded in 24-well plates and were incubated overnight. Cells were harvested by trypsinization, washed with phosphate-buffered saline (PBS), and finally centrifuged. The cell pellet was resuspended in the binding buffer and incubated with Annexin V-FITC. After that, cells were centrifuged again, washed with PBS, and resuspended in the binding buffer containing propidium iodide solution in the dark. Finally, cells were analyzed by using the approach of flow cytometry (Beckman FC400 MPL, USA). Each experiment was independently carried out in triplicate.

### Luciferase reporter assay

HEK 293T cells were purchased from the American Type Culture Collection (ATCC) and used in luciferase reporter assay. HEK 293T cells were cultured in DMEM (Gibico, Carlsbad, CA, USA). Media was supplied with 10% fetal bovine serum (Gibco, Grand Island, NY, USA), 1% penicillin/streptomycin solution (Termo Fisher Scientifc, Waltham, MA, USA). Cells were cultured in a 5% CO2 incubator at 37 °C. The 3’-untranslated regions (3’-UTR) of human MAPK1 or lncRNA MALAT1 were amplified by PCR, and individually subcloned into the pmirGLO luciferase vector (Promega, Madison, WI, USA). Site-directed mutagenesis of the miR-15b-5p binding site in MAPK1 3’-UTR and MALAT1 cDNA was conducted with a Quick change site-directed mutagenesis kit (Stratagene, La Jolla, CA, USA). For reporter assay, HEK 293T cells were co-transfected with wild-type (WT) or mutant (MUT) luciferase reporter vector and miR-15b-5p mimics using Lipofectamine 3000 (Invitrogen). Cells were harvested 48 h post-transfection and luciferase activity was revealed by Dual-luciferase Assay System (Promega) according to the manufacturer’s protocol. Renilla luciferase activity was used as a control internally.

### Cell autophagy analysis

The autophagy of cells was detected by Cyto-ID Autophagy Detection Kit (Enzo Life Sciences, NY, USA). LC3II-positive punctate pattern was observed under confocal microscope (Carl Zeiss LSM 510 META Laser Confocal Microscope, Oberkochen, German). Number of autophagosomes was counted by using the ImageJ program (Version1.48u, Bethesda, USA).

Ultrathin sections (100 nm) were cut on an ultramicrotome, counterstained with 0.3% uranium acetate and lead nitrate, and examined by a transmission electron microscope (TEM) (H7700, Hitachi, Japan).

### Animal experiments

Six-week old male ApoE^−/−^mice (C57BL/6J, n=20) were purchased from HFK bioscience company (Beijing, China) were maintained at 22 ± 2 °C, relative humidity 55% ± 5% with a 12 hours light/dark cycle. After fed with a rodent chow diet (RCD, 4.5% fat) for a week, ApoE^−/−^ mice were divided into two groups. 10 mice were fed with western diet (HFD, 21 % fat, 1.25 % cholesterol; HFK bioscience) for 12 weeks while another 10 mice were fed with rodent chow diet for 12 weeks. In different feeding groups, mice were then randomized into 2 groups (n=5 mice, respectively): antago*MALAT1*-injected and control group. The mice received tail vein injections of 25mg/kg antago*MALAT1* (GenePharma, Shanghai, China) or antagoNC once per week for 4 weeks. After fasting for 6h, mice were euthanized and blood samples from heart and aorta were collected for further analyses. All animal experiments were performed under the protocol approved by the Institutional Animal Care and Use Committee of the Central South University.

### Oil red O staining

Work at room temperature. Place the cleaned and fixed aortas in 1.5 ml Eppendorf tubes, one aorta per tube. Add 1 ml of 78 % methanol to each tube and place it on a tilted roller with gentle movement for 5 min. Replace the methanol solution and repeat this step twice. Discard the methanol and add 1 ml of fresh Oil red O solution. Incubate the tube on the tilted roller for 50–60 min. Transfer the aorta to a clean tube and wash twice with 1 ml of 78 % methanol for 5 min each on the tilted roller. Discard the methanol and refill the tube with 1 ml of PBS. If necessary, at this step aortas can be stored at 4 °C.

### Statistical analysis

All data are shown as mean ± SD. Between-group differences were assessed by Student’s *t test*; multi-group comparisons were performed by one-way Analysis of Variance (ANOVA). All statistical analyses were processed by R software. *P* < 0.05 was considered as statistically significant.

## ETHICAL APPROVAL

All procedures followed were in accordance with the ethical standards of the responsible committee on human experimentation (The First People’s Hospital of Yunnan Province, Yunnan, China) and with the Helsinki Declaration of 1964 and later versions. Informed consent to be included in the study, or the equivalent, was obtained from all patients. All institutional and national guidelines for the care and use of laboratory animals were followed.
